# Forward and inverse effects of the complete electrode model in neonatal EEG

**DOI:** 10.1152/jn.00427.2016

**Published:** 2016-11-16

**Authors:** S. Pursiainen, S. Lew, C. H. Wolters

**Affiliations:** ^1^Department of Mathematics, Tampere University of Technology, Tampere, Finland;; ^2^Newborn Medicine in the Boston Children's Hospital, Boston, Massachusetts;; ^3^Department of Engineering, Olivet Nazarene University, Bourbonnais, Illinois; and; ^4^Institute for Biomagnetism and Biosignalanalysis, University of Münster, Münster, Germany

**Keywords:** neonatal electroencephalography, skull modeling

## Abstract

This paper investigates finite element method-based modeling in the context of neonatal electroencephalography (EEG). In particular, the focus lies on electrode boundary conditions. We compare the complete electrode model (CEM) with the point electrode model (PEM), which is the current standard in EEG. In the CEM, the voltage experienced by an electrode is modeled more realistically as the integral average of the potential distribution over its contact surface, whereas the PEM relies on a point value. Consequently, the CEM takes into account the subelectrode shunting currents, which are absent in the PEM. In this study, we aim to find out how the electrode voltage predicted by these two models differ, if standard size electrodes are attached to a head of a neonate. Additionally, we study voltages and voltage variation on electrode surfaces with two source locations: *1*) next to the C6 electrode and *2*) directly under the Fz electrode and the frontal fontanel. A realistic model of a neonatal head, including a skull with fontanels and sutures, is used. Based on the results, the forward simulation differences between CEM and PEM are in general small, but significant outliers can occur in the vicinity of the electrodes. The CEM can be considered as an integral part of the outer head model. The outcome of this study helps understanding volume conduction of neonatal EEG, since it enlightens the role of advanced skull and electrode modeling in forward and inverse computations.

**NEW & NOTEWORTHY** The effect of the complete electrode model on electroencephalography forward and inverse computations is explored. A realistic neonatal head model, including a skull structure with fontanels and sutures, is used. The electrode and skull modeling differences are analyzed and compared with each other. The results suggest that the complete electrode model can be considered as an integral part of the outer head model. To achieve optimal source localization results, accurate electrode modeling might be necessary.

## Abstract (precis)

The effect of the complete electrode model on electroencephalography forward and inverse computations is explored. A realistic neonatal head model, including a skull structure with fontanels and sutures, is used. The electrode and skull modeling differences are analyzed and compared with each other. The results suggest that the complete electrode model can be considered as an integral part of the outer head model. To achieve optimal source localization results, accurate electrode modeling might be necessary.

this paper explores the mathematical complete electrode model (CEM) (Cheng et al. 1989; Ollikainen et al. 2000; Pursiainen 2012; Somersalo et al. 1992; Vallaghé et al. 2009; Vauhkonen 1997) in neonatal electroencephalography (EEG) forward simulation (Baillet et al. 2001; de Munck et al. 2012; Gargiulo et al. 2015; Lew et al. 2013; Niedermeyer and da Silva 2004). Our objective is to evaluate the differences between the CEM and the standard point electrode model (PEM) for a head of a 3-day-old neonate for which the electrode diameter is unusually large compared with that of the head. The CEM consists of a set of boundary conditions originally developed for impedance tomography (Cheney et al. 1999) in which the electrodes are used to inject currents and measure voltage data simultaneously, thus necessitating accurate control of impedances and surface currents to minimize forward simulation errors.

In EEG, the net currents flowing through the electrodes are zero. Subelectrode surface currents due to potential variation, however, exist. These shunting effects consume a small part of the electric field energy, leading to lower voltage amplitudes than what is predicted by a mathematical model not including the skin-electrode interaction. The shunting is more pronounced the larger the relative electrode size (angular coverage) or the lower the contact impedance is (Ollikainen et al. 2000). The first aspect of these makes the neonatal EEG interesting as a target of investigation. The second one is important from the viewpoint of choosing an optimal measurement impedance: the general rule of thumb is that decreasing the impedance improves the signal amplitude, because of better power transfer between the skin and the electrode, until around 100–500 Ohm is reached. Below 100 Ohm, shunting increases significantly, altering the electrode voltage and the surface potential distribution (American Clinical Neurophysiology Society 2006; Duffy et al. 2012).

The CEM accounts for more accurate modeling of electrode shunting and thereby, in principle, extends the interval of applicable impedances beyond that of the classical PEM. In the CEM, the voltage experienced by an electrode is the integral average of the potential distribution below its contact surface, whereas the PEM relies on a single mesh node. This study aims to find out how the electrode voltage predicted by these two models differs, when 10-mm-diameter electrodes are used in combination with a newborn head. Additionally, the voltage values and variation on electrode surfaces are investigated using the following two source locations: *1*) next to the C6 (5th) electrode and *2*) directly under the Fz electrode and the frontal fontanel. Because of the significant differences between a newborn and a normal adult skull, this study uses a realistic neonatal head model including a skull with fontanels and sutures (Darbas M, Diallo El Badia AM, Lohrengel S, unpublished observations; Lew et al. 2013). As the primary forward simulation method, we use the finite element method (FEM) (Braess 2001; de Munck et al. 2012; Lew et al. 2009), which is a flexible alternative to the boundary element method (Acar and Makeig 2010; Gramfort et al. 2011; Mosher et al. 1999; Stenroos and Sarvas 2012) as well as to finite difference and volume methods (Cook and Koles 2006; Montes-Restrepo et al. 2014; Vatta et al. 2009; Wendel et al. 2008). Particularly important for our study is that a FEM mesh can be simultaneously adapted to both boundary and interior structures of the computational domain. Our CEM implementation is mathematically rigorous: the lead field matrix describing the forward simulation is constructed directly based on the weak form of the electric potential (Poisson) equation (Braess 2001). Moreover, the source current distribution is modeled via the Whitney (Raviart-Thomas)-type vector basis functions, with divergence satisfying square integrability condition (Bauer et al. 2015; Pursiainen 2012; Pursiainen et al. 2011; Tanzer et al. 2005).

This study includes numerical experiments in which a wide range of electrode impedances from 0.1 Ohm to 12 kOhm are investigated. The differences of the electrode voltages predicted by CEM and PEM are explored using a descriptive statistical approach in which the relative difference and magnitude measures (RDM and MAG) are analyzed via box plots for five different source eccentricities (relative norms). Additionally, voltages and voltage variation on electrode surfaces with two different electrode impedance values are visualized. The inverse aspect of the forward simulation differences is explored through the minimum current estimation (MCE) technique (Calvetti et al. 2009; Lucka et al. 2012; Uutela et al. 1999).

Based on our results, the CEM can be considered as an integral part of the head model. The median level of the RDM and MAG between CEM and PEM was found to be small, around 0.7 and 0.5%, respectively, regarding the impedances above the commonly recommended lower limit of 100 Ohm. However, the dependence of these differences on the source position was found to be significant: the spread of the results extended considerably toward the electrodes with outliers up to 23 and 36% occurring in their vicinity.

## MATERIALS AND METHODS

### Forward Model

Let *e*_*ℓ*_, *ℓ* = 1, 2,  . . . ,* L* (*L* = the number of electrodes) denote the skin-electrode contact surfaces on the boundary ∂Ω of the head Ω. Given the primary current density ${\overset{\to }{j}}^{p}$ and a conductivity tensor σ within Ω, the resulting electric potential field *u* within Ω can be obtained as a solution of the quasistatic electric potential equation

(1) $$\nabla \cdot \left(\sigma \nabla u\right)-\nabla \cdot {\overset{\to }{j}}^{p}$$

with the following CEM boundary conditions (Cheng et al. 1989):

(2) $$\sigma {\nabla u}_{}\cdot \overset{\to }{n}|{}_{\partial \Omega \backslash e_{\ell }}=0,$$

(3) $$\int_{e_{\ell }}\sigma \nabla_{}u\cdot \overset{\to }{n}dS=0,$$

(4) $$\left(u+{\overset{˜}{Z}}_{\ell }\sigma {\nabla u}_{}\cdot \overset{\to }{n}\right)|{}_{e_{\ell }}=U_{\ell ,}\;for\;\ell =1,2,\ldots ,L.$$

The first one of these requires that the normal current −σ▽*u* ⋅ $\overset{\to }{n}$ on ∂Ω can flow out of or into the domain only through electrodes. The second one ensures that the net current flowing through each electrode is zero, and the third one defines the potential jump on the skin-electrode contact interface. The potential jump is proportional to the normal current density and to the pointwise effective skin-electrode contact impedance *Z̃*_*ℓ*_ with the units Ohm per square meter. The voltage of the *k*th electrode is denoted by *U*_*ℓ*_. For simplicity, we assume that *Z̃*_*ℓ*_ is of the form *Z̃*_*ℓ*_ = *Z*_*ℓ*_*A*_*ℓ*_ where *Z*_*ℓ*_ is the average impedance (Ohm) of the contact surface area *A*_*ℓ*_ (m^2^). Under this assumption, the *ℓ*th electrode voltage is given by the integral mean *U*_*ℓ*_ = (1/*A*_*ℓ*_)$\underset{e_{\ell }}{\int }udS\;$.

#### Weak form.

The weak form (Somersalo et al. 1992; Vauhkonen 1997) of *Eqs. 1–4*, i.e., the CEM, can be written as follows (appendix):

(5) $$-\underset{\Omega }{\int }\left(\nabla \cdot {\overset{\to }{j}}^{p}\right)v\;dV=\underset{\Omega }{\int }\sigma {\nabla u}_{}\cdot {\nabla v}_{}\;dV+\overset{L}{\underset{\ell =1}{\sum }}\frac{1}{Z_{\ell }A_{\ell }}\underset{e_{\ell }}{\int }uv\;dS-\overset{L}{\underset{\ell =1}{\sum }}\frac{1}{Z_{\ell }A_{\ell }^{2}}\underset{e_{\ell }}{\int }u\;dS\underset{e_{\ell }}{\int }v\;dS.$$

Here, d*V* and d*S* denote differentials with respect to volume and surface, respectively. If the divergence of ${\vec{j}}^{p}$ is square integrable, i.e., if it holds that ${\vec{j}}^{p}$ ∈ {$\vec{w}$|▽ ⋅ $\overset{\to }{w}$ ∈ *L*^2^(Ω)}, then *Eq. 5* has a unique solution *u* ∈ *H*^1^(Ω) = {*w* ∈ *L*^2^(Ω)|∂*w*/∂*x*_*i*_ ∈ *L*^2^(Ω), *i* = 1,2,3} satisfying *Eq. 5* for all *v* ∈ *H*^1^(Ω). In this study, we assume that *u* and *v* belong to a subspace S ⊂ *H*^1^(Ω) spanned by finite-element basis functions. If the support of the *l*th electrode tends to one point ${\overset{\to }{p}}_{\ell }$, i.e., if *e*_*ℓ*_ $\to {\overset{\to }{p}}_{\ell }$, then the following relation of the form (1/*A*_*ℓ*_) $\underset{e_{\ell }}{\int }udS\;\to \;u$ (${\overset{\to }{p}}_{\ell }$) holds for *u*, *v*, *uv*, and *U*_*l*_, and consequently, the CEM weak form tends to PEM one, i.e.,

(6) $$-\underset{\Omega }{\int }\left(\nabla \cdot {\overset{\to }{j}}^{p}\right)v\;dV=\underset{\Omega }{\int }\sigma {\nabla u}_{}{\cdot \nabla v}_{}\;dV$$

### FEM Forward Simulation

Given function bases ψ_1_,ψ_2_, . . . ,ψ_*N*_ ∈ *H*^1^(Ω) and ${\vec{w}}_{1}$,${\overset{\to }{w}}_{2}$, . . . ,$\vec{w}$_*M*_ ∈ {$\vec{w}$|▽ ⋅ $\vec{w}$ ∈ *L*^2^(Ω)} associated with the finite element (FE) mesh *T*, the potential and primary current density can be approximated as the following finite sums $u_{T}=\sum_{i=1}^{N}z_{i}\psi_{i}$ and ${\overset{\to }{j}}_{T}^{p}=\overset{M}{\underset{i=1}{\sum }}x_{i}{{\overset{\to }{w}}_{i}.}_{}$ Denoting *z* = (*z*_1_, *z*_2_, . . . , *z*_*N*_) the weak form *Eq. 5* within the subspace of the basis functions is given by

(7) $$\left(\begin{array}{cc} A & -B\\ -B^{T} & C \end{array}\right)\left(\begin{array}{l} \begin{array}{c} z \end{array}\\ v \end{array}\right)=\left(\begin{array}{c} \begin{array}{l} -Gx\\ 0 \end{array} \end{array}\right)$$

Matrix **A** is of the form

(8) $$a_{i,j}=\underset{\Omega }{\int }\sigma \nabla {\psi {}_{i}\cdot \nabla \psi }_{j}\;dV+\overset{I}{\underset{l=1}{\sum }}\underset{e_{l}}{\int }\psi_{i}\psi_{j}\;dS,$$

where additionally *a*_*i*′, *j*_ = ∂_*i*′, *j*_ with ∂_*i*′,*i*_ = 1 and ∂_*i*′, *j*_ = 0 for *i*′ ≠ *j* (Kronecker delta) for a single nodal basis function ψ_*i*_ attaining its maximum on the part of the boundary not covered by the electrodes. The entries of ***B*** (N-by-L), ***C*** (L-by-L), and ***G*** (N-by-M) are given by

(9) $$b_{i,\ell }=\frac{1}{Z_{\ell }A_{\ell }}\underset{e_{\ell }}{\int }\psi_{i}\;dS,c_{i,\ell }=\frac{\delta_{i,\ell }}{Z_{\ell }}\;and\;g_{i,k}=\underset{\Omega }{\int }\left(\nabla \cdot {\overset{\to }{w}}_{k}\right)\psi_{i}\;dV$$

Electrode voltages *y* = (*U*_1_,*U*_2_, . . . ,*U*_*L*_) predicted by *Eq. 7* can be obtained via *y* = *Rv* in which multiplication by the real L-by-L matrix ***R***, *r*_*ℓℓ*_ = 1 − 1/*L* for *ℓ* = 1,2, . . . ,*L* and *r*_*i*,*ℓ*_ = −1/*L* for *i* ≠ *ℓ*, fixes the sum of the electrode voltages to zero. Consequently, the potential data predicted by the forward model can be simulated as given by *y* = *Lx*, where the lead-field matrix is of the following form ***L*** = ***R***(***B***^*T*^***A***^−1^***B*** − ***C***)^−1^***B***^*T*^***A***^−1^***G***. To determine the lead-field matrix ***L*** efficiently, the matrix ***H*** = ***B***^*T*^***A***^−1^ is first computed using iterative solvers for ***AH***^*T*^ = ***B***^*T*^. When the PEM is in question, i.e., when *e*_*ℓ*_ → ${\overset{\to }{p}}_{\ell }$, the lead-field matrix tends to ***L*** = ***RB***′^T^***A***′^−1^***G*** with $b_{i,\ell }^{\prime }$ = ψ_*i*_(${\overset{\to }{p}}_{\ell }$) and

(10) $$a_{i,j}^{\prime }=\underset{\Omega }{\int }\sigma \nabla \psi_{i}\cdot \nabla \psi_{j}\;dV\;\;with\;\;a_{i^{\prime },j}=\psi_{i^{\prime },j}$$

following from *Eq. 6*.

### Synthetic Dipoles

**Fig. 1.**
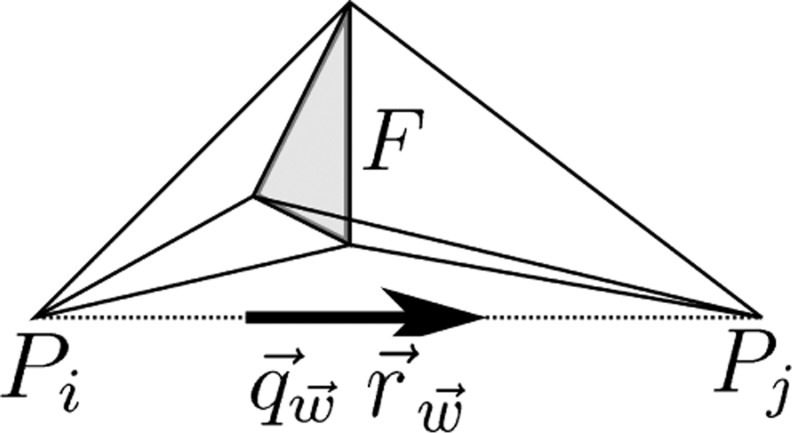
A schematic illustration of the synthetic dipole corresponding to the face *F*. The position $\vec{r}$ and dipole moment ${\vec{q}}_{w}$ are determined by the midpoint and directional unit vector of the line segment between *P*_*i*_ and *P*_*j*_, respectively (Bauer et al. 2015).

The primary current and electric potential fields were modeled using a Whitney (Raviart-Thomas) and linear Lagrangian (nodal) function basis, respectively. This combination yields a simple model of synthetic dipoles (Bauer et al., 2015; Pursiainen et al., 2011; Pursiainen, 2012; Tanzer et al., 2005) with dipole moment and position determined by

(11) $${\overset{\to }{q}}_{w}=\frac{{\overset{\to }{r}}_{P_{j}}-{\overset{\to }{r}}_{P_{i}}}{\left\|{\overset{\to }{r}}_{P_{j}}-{\overset{\to }{r}}_{P_{i}}\right\|}\;and\;{\overset{\to }{r}}_{w}=\frac{1}{2}\left({\overset{\to }{r}}_{P_{j}}+{\overset{\to }{r}}_{P_{i}}\right),$$

where ${\overset{\to }{r}}_{P_{j}}$ and ${\overset{\to }{r}}_{P_{i}}$ are the position vectors of mesh nodes *P*_*j*_ and *P*_*i*_ on the opposite sides of the common face *F* in an adjacent pair of tetrahedra (Fig. 1). The synthetic dipole moment is given by the integral ${\overset{\to }{q}}_{w}=\underset{}{\int_{\Omega }}\overset{\to }{w}dV$ and the position is the midpoint of the nodes *P*_*j*_ and *P*_*i*_ for which

(12) $$G_{\psi ,w}=\underset{\Omega }{\int }\psi \left(\nabla \cdot \overset{\to }{w}\right)dV=\frac{S_{\psi ,P_{j}}-S_{\psi ,P_{i}}}{\left\|{\overset{\to }{r}}_{P_{j}}-{\overset{\to }{r}}_{P_{i}}\right\|}$$

with

(13) $$S_{\psi ,P}=\left\{ \begin{array}{l} 1,\;if\;\psi \;corresponds\;to\;P,\\ 0,\;otherwise \end{array}\right.$$

*Equations 11* and *12* can be proven via straightforward calculation as shown, e.g., by Bauer et al. (2015).

### Minimum Current Estimation

The inverse aspect of modeling differences was explored via the MCE approach (Uutela et al. 1999) that minimizes the *ℓ*^1^-regularized function

(14) $$f\left(x|y\right)={\left\|Lx-y\right\|}_{2}^{2}+\gamma {\left\|x\right\|}_{1},$$

where γ > 0 is a regularization parameter. The minimizer was estimated using the following iterative alternating sequential (IAS) algorithm (Calvetti et al. 2009):

*1*. Set *i* = 0 and *x*_0_ = (1,1, . . . ,1).
2. Find *x*_*i*+1_ as the least-squares solution of the linear system
  (15) $$\lfloor \begin{array}{c} L\\ \gamma^{1/2}D_{x_{i}}^{-1/2} \end{array}\rfloor x_{i+1}=\left[\begin{array}{l} \begin{array}{c} y \end{array}\\ 0 \end{array}\right],$$

where *D*_*x*_ = diag(|*x*_1_|, |*x*_2_|, . . . ,|*x*_*m*_|), and set *i* → *i* + 1.

3) If *i* is less than the desired number of iterations, repeat step number 2.
4) Set *x* = *x*_*i*_ as the final estimate.

The result is a minimally supported current distribution *x* (appendix) that is here interpreted as a single dipole. Based on *x*, estimates of dipole location and direction were obtained as the weighted average

(16) $$\overset{\to }{r}=\frac{\sum_{i=1}^{n}\left|x_{i}\right|{\overset{\to }{r}}_{w_{i}}}{\sum_{i=1}^{n}\left|x_{i}\right|},\;\;\;\;\;\;\;\overset{\to }{q}=\frac{\sum_{i=1}^{n}\left|x_{i}\right|{\overset{\to }{q}}_{w_{i}}}{\sum_{i=1}^{n}\left|x_{i}\right|}.$$

### Numerical Experiments

**Fig. 2.**
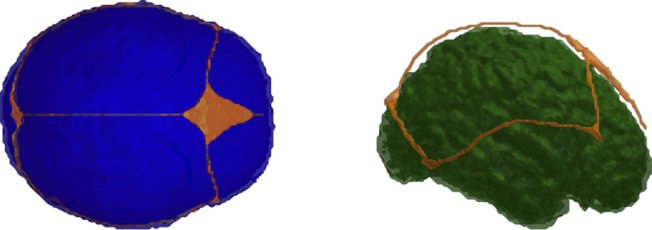
The neonatal head model. *Left*, axial view of the skull (blue) together with fontanel/suture (orange). *Right*, sagittal view of the grey matter (green) and fontanel/suture (orange).

Our numerical experiments used three variations (I-III) of a neonate head model that were created based on the segmentation of T1-weighted MPRAGE images of a 3-day-old healthy infant (Lew et al. 2013). The segmentation was conducted manually in FreeView, a volume and surface visualization tool within the FreeSurfer software application (Dale et al. 1999), resulting in segmented skull, scalp, cerebrospinal fluid, grey and white matter, and anterior fontanel compartments. Other fontanels, including the posterior, sphenoidal, and mastoid fontanels, were segmented based on the knowledge of the skull geometry that was generated for a neonate (Fig. 2).

*Model I* was the gold standard with the following five tissue compartments and values of conductivity (Table 1): the brain (grey and white matter) 0.33 S/m (Haueisen 1996), cerebrospinal fluid (CSF) 1.79 S/m (Baumann et al. 1997), bone 0.04 S/m and fontanel/suture stuctures (openings) within the skull 0.3 S/m (Lew et al. 2013), and skin 0.33 S/m (Haueisen 1996). *Model II* was otherwise identical to *model I* except that the conductivity of fontanel/suture (0.3 S/m), characteristic to a neonatal head, was replaced with that of bone (0.04 S/m), resulting in a closed skull. *Models I* and *III* differed with respect to the bone conductivity, which in *model III* was given the value 0.1 S/m. The voltage data were obtained using a cap of 74 circular electrodes with 10 mm diameter (10–20 system). Each electrode was formed as a set of surface triangles with the center point within 5 mm distance from the electrode center. In the visualization of the results, the nearest point interpolation method between the original and a refined surface mesh was used. Fourteen different impedance values, equally distributed on a logarithmic scale, were used to cover a range of values between 0.10 Ohm and 12 kOhm average contact impedance (ACI).

**Table 1.** Model-specific conductivities of the brain (grey and white matter) (Haueisen 1996), CSF (Baumann et al. 1997), bone, fontanel/suture stuctures (openings) within the skull (Lew et al. 2013), and skin (Haueisen 1996)

Model	Scalp	Skull	Fontanel/Suture	CSF	Brain
I	0.33	0.04	0.3	1.79	0.33
II	0.33	0.04	0.04	1.79	0.33
III	0.33	0.1	0.3	1.79	0.33

CSF, cerebrospinal fluid.

The relative difference and magnitude (RDM and MAG) measures

(17) $$RDM\left(u_{1},u_{2}\right)=\frac{100}{2}{\left\|\frac{u_{1}}{{\left\|u_{1}\right\|}_{2}}-\frac{u_{2}}{{\left\|u_{2}\right\|}_{2}}\right\|}_{2},$$

(18) $$MAG\left(u_{1},u_{2}\right)=100\frac{{\left\|u_{2}\right\|}_{2}}{{\left\|u_{1}\right\|}_{2}}-100$$

between two voltage predictions *u*_1_ vs. *u*_2_ were evaluated. RDM approximates topography changes and MAG the difference in magnitude between two forward solutions. The electrode/head model comparisons investigated are listed in Table 2. *Comparison 1* is the primary one, and *comparisons 2* and *3* are used as a reference to show the voltage differences obtained with the open (I) and closed (II) skull model as well as the effect of alternative skull conductivity (III). The results were visualized via box plots showing the median, spread (IQR), i.e., the centermost (thickened) interval containing 50% of the sample points, as well as the (narrow) interval between the minimal and maximal result.

**Table 2.** Comparisons (1–3) investigated in the numerical experiments

Number	Electrodes 1	Head 1	Electrodes 2	Head 2
1	CEM	I	PEM	I
2	CEM	I	CEM	II
3	CEM	III	PEM	III

CEM, complete electrode model; PEM, point electrode model.

Further comparison between CEM (I) and PEM (I) concerned random samples of 100 random sources collected at five different source depths in the brain compartment with eccentricities (relative norms) 0.2, 0.4, 0.6, 0.8, and 0.98. In addition to evaluation of RDM and MAG, the position, angular (degree), and norm (depth) difference (PD, AD, and ND, respectively) with

(19) $$PD\left(r_{w},\overset{\to }{r}\right)={\left\|{\overset{\to }{r}}_{w}-\overset{\to }{r}\right\|}_{2},$$

(20) $$AD\left({\overset{\to }{q}}_{w},\overset{\to }{q}\right)=\arccos\left(\frac{{\overset{\to }{q}}_{w}\cdot \overset{\to }{q}}{{\left\|{\overset{\to }{q}}_{w}\right\|}_{2}{\left\|\overset{\to }{q}\right\|}_{2}}\right),$$

(21) $$ND\left({\overset{\to }{r}}_{w},\overset{\to }{r}\right)={\left\|{\overset{\to }{r}}_{w}\right\|}_{2}-{\left\|\overset{\to }{r}\right\|}_{2},$$

${\vec{r}}_{w}$ and $\vec{r}$ as in *Eqs. 11* and *16*, respectively, were analyzed via source localization analysis in which each one of the sources was reconstructed with the IAS algorithm. For each source, the exact voltage data were calculated using the CEM, and the reconstruction was computed via PEM forward simulation. The synthetic dipole used in generating the data was not included in the reconstruction procedure to avoid the inverse crime condition, i.e., overly optimistic data fit.

The number of IAS iteration steps was chosen to be 50, and the regularization parameter γ was given the value 1.4 × 10^−6^. The present choice of γ can be motivated through a correspondence to a hierarchical Bayesian probability model with a conditionally Gaussian prior and a gamma hyperprior (Calvetti et al. 2009; Lucka et al. 2012). Assuming that the measurements contain Gaussian white noise with very low SD *ν* = 0.001 and setting the scaling (initial prior variance) and shape parameter of the gamma density to θ_0_ = 1 and β = 1.5, then the resulting posterior probability density can be maximized via the IAS algorithm by setting γ = *ν*^2^√2/θ_0_ equal to 1.4 × 10^−6^ (Calvetti et al. 2009).

Additionally, electrode voltages and voltage variation *u* − U_*ℓ*_ underneath the electrodes *ℓ* = 1,2, . . . ,*L* were analyzed for two 10-nAm sources (A and B) placed next to the C6 (5th) electrode and directly under the Fz electrode and the frontal fontanel, respectively. The voltages corresponding to *sources A* and *B* were produced using the following electrode/head model and impedance combinations: PEM (I), CEM (I) 2.0 kOhm, CEM (I) 0.1 Ohm, and CEM (II) 2.0 kOhm.

## RESULTS

**Fig. 3.**
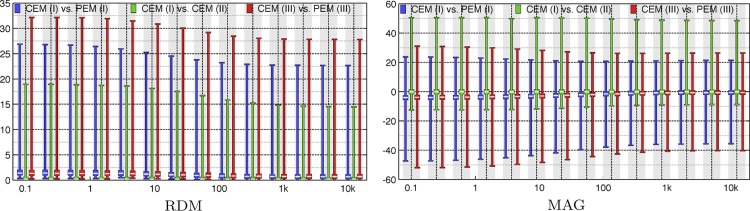
The distributions of relative difference measure (RDM, *left*) and magnitude measure (MAG, *right*) for the electrode/head model *comparisons 1* complete electrode model (CEM) (I) vs. point electrode model (PEM) (I), *2*) CEM (I) vs. CEM (II), and *3*) CEM (III) vs. PEM (III) covering the tested range of impedances from 0.1 Ohm to 12 kOhm. The horizontal axis has a logarithmic scaling and unit Ohm. The unit of the vertical axis is %.

**Fig. 4.**
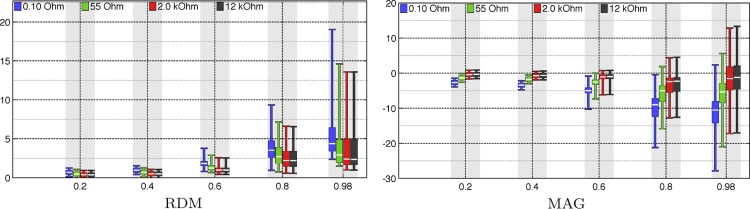
Eccentricity analysis for *comparison 1*: RDM (*left*) and MAG (*right*) for different eccentricities (relative norms) 0.2, 0.4, 0.6, 0.8, and 0.98 (horizontal axis). Each bar corresponds to a sample of 100 random sources. The unit of the vertical axis is %.

**Fig. 5.**
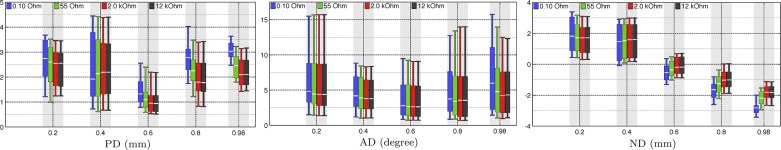
Eccentricity analysis for *comparison 1*: position difference (PD, *left*), angular difference (AD, *middle*), and norm difference (ND, *right*) visualized for eccentricities (relative norms) 0.2, 0.4, 0.6, 0.8, and 0.98 (horizontal axis). Each bar corresponds to a sample of 100 random sources. The unit of the vertical axis is mm.

**Fig. 6.**
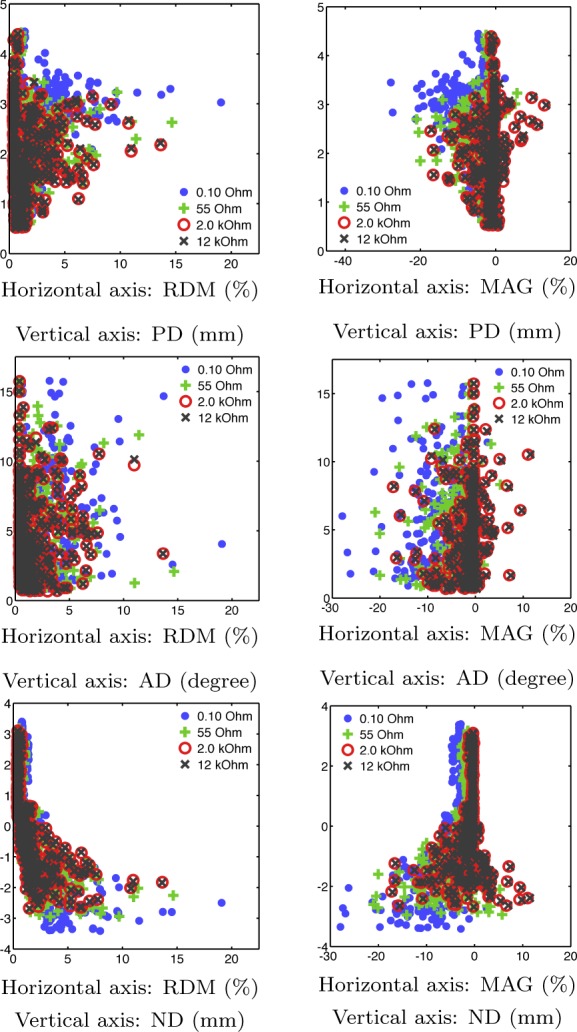
PD/AD/ND plotted against RDM/MAG suggests that large values in the absolute value of RDM/MAG correlate with those of PD/AD/ND. Outliers in RDM/MAG tend to result in large deviations of PD/AD/ND. Each point cloud includes all of the results obtained for a given impedance value and eccentricities (relative norms) 0.2, 0.4, 0.6, 0.8, and 0.98.

**Fig. 7.**
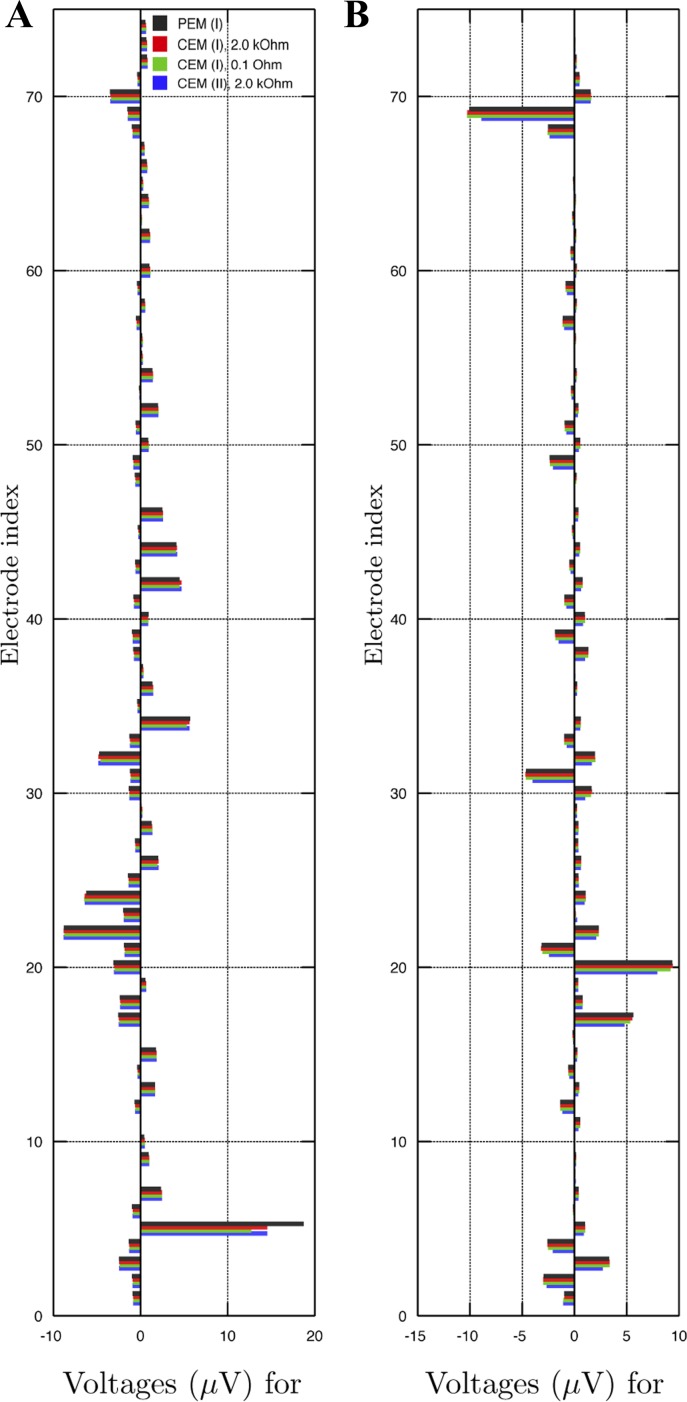
Voltage variation for the two 10-nAm sources (*A* and *B*). The most significant variation occurs on the electrodes near the source. The variation can be observed to diminish along with the impedance because of increasing shunting effects: for 0.1 Ohm impedance, it is close to zero.

**Fig. 8.**
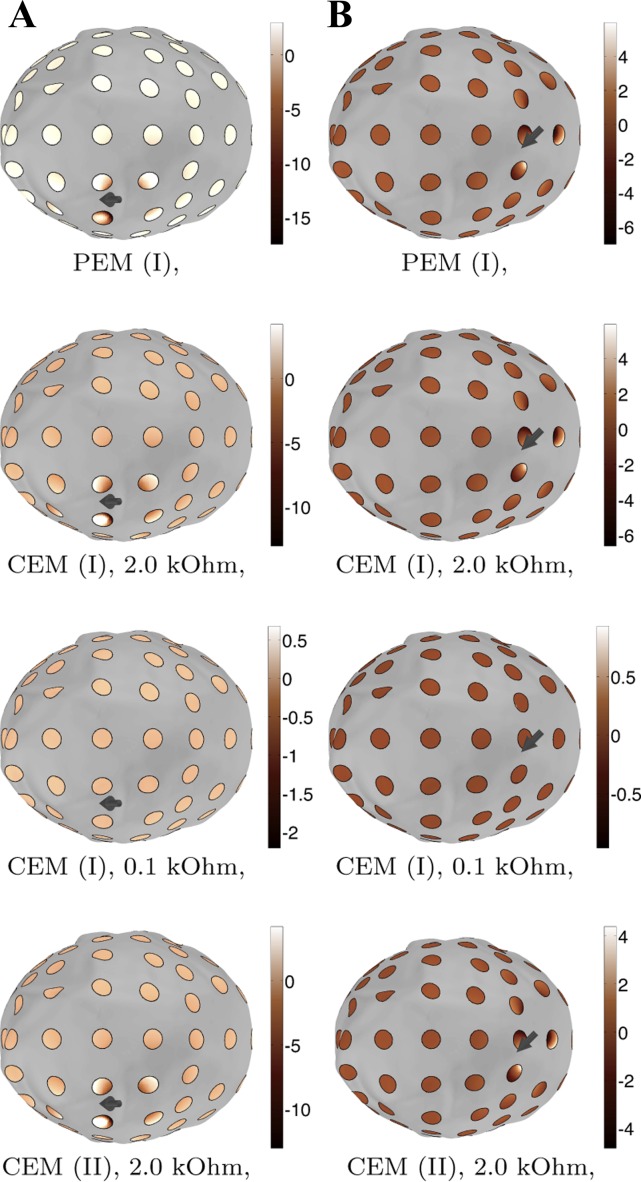
The voltage data for *sources A* and *B* located next to the C6 (5th) electrode and below the Fz electrode and the frontal fontanel, respectively. In the case of *source A* (*A*), PEM (I) produces an outlier at the C6 electrode. Also in the case of *source B* (*B*), PEM (I) yields significantly different values compared with other techniques for electrodes close to the source, e.g., AFz and F2 (20th and 69th) electrodes.

The results have been included in Figs. 3–8. Of these, Fig. 3 includes *comparisons 1–3*. Eccentricity analysis for *comparison 1* can be found in Figs. 4–6. RDM, MAG, PD, AD, and ND are shown in Figs. 4 and 5, and the dependence between RDM/MAG and PD/AD/ND is shown in Fig. 6. Finally, Figs. 7 and 8 visualize the electrode voltages and subelectrode potential variation for *sources A* and *B*, respectively.

In *comparison 1*, the median RDM and MAG vary from 1.5 to 0.7% (RDM) and −3.5 to −0.5% (MAG) when moving from 0.1 Ohm to 12 kOhm. The maximal (absolute) value decreases from 27 to 23% (RDM) and from 48 to 36% (MAG), respectively. Based on both RDM and MAG, the difference distributions stay virtually unchanged between 100 Ohm and 12 kOhm. The point of the steepest slope is located between 10 and 100 Ohm. In *comparisons 1* and *3*, the median and spread of both RDM and MAG coincide up to the accuracy of 0.2, whereas in *comparison 2* those appear to be on a lower level.

Figure 4 analyzes the results of *comparison 1* with respect to the source depth for eccentricities. Each bar visualizes a distribution of 100 random sources. The absolute values of both RDM and MAG can be observed to grow along with the eccentricity. Close to the surface (eccentricity 0.98), the medians of RDM and MAG were found to vary between 2–4% and −1 to −11%, respectively.

PD, AD, and ND calculated for *comparison 1* were observed to increase in absolute value toward the surface of the head (Fig. 5). At 0.98 eccentricity, the median PD was 2.1–3.0 mm and the median AD 4–6 degrees. As indicated by the negative median ND of −2.8 to −1.8 mm, the sources were localized deeper than they actually were. ND and PD are close in magnitude, suggesting that the localization difference is mainly in the normal direction. Figure 6 suggests that large values in the absolute value of RDM/MAG correlate with those of PD/AD/ND in a nonsystematic way.

Figure 7 shows voltage patterns for *sources A* and *B* as obtained with the modeling techniques PEM (I), CEM (I) 2.0 kOhm, CEM (I) 0.1 Ohm, and CEM (II) 2.0 kOhm. In the case of *source A*, PEM (I) produces an outlier at the C6 electrode, whereas the other techniques result in mutually similar patterns. In the case of *source B*, CEM (II) 2.0 Ohm yields ∼1–2 μV lower voltage amplitudes than the other techniques for the AFz and F2 (20th and 69th) electrode which are close to the source. Figure 8 shows the voltage variation beneath the electrodes for *sources A* and *B*, showing that the incorrect approximation obtained with PEM (I) for *source A* at the 5th electrode appears to be due to the difference between the electrode center point and integral average values, and that the magnitude of the variation shrinks along with the impedance value.

## DISCUSSION

Our numerical experiments focused on forward simulation differences between the CEM and the PEM boundary conditions with a realistic neonatal head model as the target domain. These experiments were motivated by the small size of the head compared with which the typical electrode diameter of 10 mm is large. Consequently, the differences can be expected to be larger than in a normal adult head. To investigate these changes, we investigated the relative difference and magnitude measures (RDM and MAG) for electrode impedances between extremely low 0.1 Ohm and comparably high 12 kOhm as well as for five different source eccentricities. MCE-based source localization was used to enlighten the effect of forward simulation differences and also visual comparisons of the voltage patterns, and subelectrode potential distributions were presented.

The results suggest that the median level of RDM and MAG appears to be rather small, with 0.5–3.5% for all impedances. The outliers were significant in the vicinity of the electrodes throughout the interval of tested impedances, extending in absolute value to 23 and 36% for RDM and MAG, respectively, for the normally used range of over 100 Ohm. Hence, it is obvious that the CEM can improve the forward simulation accuracy in cortical regions. Above 100 kOhm the impedance value has a negligible effect on RDM and MAG. The exact value of impedance might thus be of minor importance in applications of experimental EEG with respect to the forward simulation accuracy. Based on the MAG, it is obvious that the PEM led to systematically lower amplitudes than the CEM due to the absence of the shunting effects.

As a whole, the differences between CEM and PEM (*comparison 1*) were found to be similar in terms of descriptive statistics, to those between the open (I) vs. closed (II) skull model (*comparisons 2* and *3*). In both cases large values of comparable magnitude were observed in the vicinity of the skull. In the former case, these outliers occur close to the electrodes, and, in the latter one, close to the openings, i.e., the fontanels and sutures. Because of the difficulty of creating a point cloud without a bias in either of these regions, the eccentricity dependence was analyzed only for *comparison 1*, which was of primary interest. The CEM-PEM differences were also observed to increase along with the conductivity of the skull tissue based on the results obtained with 0.04 and 0.1 S/m of (I) and (III). Hence, the CEM can be considered as an integral part of the outer head model akin to accurate skull modeling (Lew et al. 2013).

The MCE results suggest that the differences between the CEM and PEM can be reflected in terms of source localization results. An increase in RDM and MAG between CEM and PEM leads to slightly larger localization differences PD, AD, and ND. With respect to impedance, marginal deterioration of the localization accuracy was observed below 100 Ohm. Above that value, the median PD was maximally 2.5 mm, which is close to the real extent of the neural sources (de Munck et al. 2012). The median ND was below zero close to the electrodes, suggesting that PEM-based inverse estimates tend to localize sources 1–2 mm deeper than they actually are. This is obviously connected to the absence of the shunting effects in PEM, which is why the actual voltage magnitude is, in general, lower than what is predicted by the PEM. The general level of AD was significant, which is at least partially due to the MCE inversion strategy being nonoptimal with respect to finding the direction. However, the distribution of AD was similar to that of PD regarding the eccentricity and impedance, showing that, also with respect to AD, the accuracy of the MCE inversion decreases as the source modeling differences go up. MCE was chosen as the inverse approach, since it does not need the a priori knowledge of the number of underlying sources and can thus often better be used to recover brain activity in a real application context.

To assure the reliability of the results, a realistic head model and an advanced source modeling technique were used in the numerical experiments. The accuracy of the applied Whitney type model has been recently shown to be slightly superior to the classical St. Venant (Buchner et al. 1997; Schönen et al. 1994; Toupin 1965) and Partial Integration (Awada et al. 1997; Weinstein et al. 2000; Yan et al. 1991) approaches at the orientations and positions of the synthetic dipoles and between these two, slightly inferior to St. Venant, if arbitrary sources are in question (Bauer et al. 2015). Nodes connected to the surface of the brain were excluded from the forward simulation to eliminate outliers due to material parameter discontinuities (de Munck et al. 2012). The head model involves some uncertainty, since a neonatal head changes dramatically within months after birth, and especially changes in the skull compartment have significant impact on the EEG forward problem (Dannhauer et al. 2011; Lew et al. 2009). In particular, rapid ossification quickly decreases the conductivity of the skull, a higher value 0.1 S/m was tested in addition to 0.04 S/m (Lew et al. 2013). An increase in the conductivity of the skull was observed to slightly strengthen the differences between CEM and PEM. It is noteworthy that a recent study (Gargiulo et al. 2015) suggests than an even higher skull conductivity could be considered, which would lead to even higher errors.

Significant for clinical neonatal studies is the finding that the electrode and skull modeling errors might be comparable to each other. It has been shown that the neonatal unossified skull structure allows improved source localization compared with an adult EEG because of the higher focality of dipolar EEG patterns due to higher skull conductivity (Odabaee et al. 2014). To achieve optimal source localization results, accurate electrode modeling might be necessary. An important aspect regarding clinical applications is also that the CEM can be implemented in a straightforward way via the approach presented in this paper. It can be easily included to any EEG forward solver by associating an electrode with a set of elements instead of a single point and by forming the boundary condition *matrixes B* and *C* that are not present in the PEM. Because it seems that the CEM can improve the modeling accuracy compared with the PEM, the CEM is a well-motivated part of the forward solver.

To conclude our study, we propose that the CEM can improve EEG forward simulation accuracy. Yet, it is not a completely necessary feature in source modeling of standard EEG measurements, where the impedance is >100 Ohm. In our future research, the CEM may be used with combined transcranial current stimulation (tCS) (Breitling et al. 2016; Dannhauer et al. 2012; Eichelbaum et al. 2014; Guler et al. 2016b, 2016a; Herrmann et al. 2013) and EEG, in which the CEM can be used for both stimulation and measurement electrodes. As motivated by the current results, numerical tests could be performed over a range of skull sizes and parameter values. An important topic would be also to study the CEM using experimental data to validate the present numerical results in practice. A well-controlled phantom (Liehr and Haueisen 2007; Wetterling et al. 2009), or possibly even more informative, animal model study (Lau et al. 2016) should be one of the next steps in the further evaluation of the CEM. In work by Lau et al. (2016), the important first steps have been carried out in characterizing errors in source reconstruction due to ignoring skull defects and in assessing the ability of an exact FE head model to eliminate such errors in an evaluation study with an anesthetized rabbit.

## GRANTS

S. Pursiainen was supported by the Centre of Excellence in Inverse Problems and Key Project no. 305055 of the Academy of Finland. S. Lew was supported by National Institute of Biomedical Imaging and Bioengineering Grants R01-EB-0009048 and R21-EB-008547 and National Science Foundation Grant 0958669. C. H. Wolters was supported by the Deutsche Forschungsgemeinschaft (DFG) project WO1425/7-1, by the DFG Priority Program 1665 project WO1425/5-2, and by the European Union project ChildBrain (Marie Curie Innovative Training Networks, no. 641652).

## DISCLOSURES

No conflicts of interest, financial or otherwise, are declared by the authors.

## AUTHOR CONTRIBUTIONS

S.P., S.L., and C.H.W. conceived and designed research; S.P. performed experiments; S.P. analyzed data; S.P., S.L., and C.H.W. interpreted results of experiments; S.P. prepared figures; S.P. and S.L. drafted manuscript; S.P., S.L., and C.H.W. edited and revised manuscript; S.P., S.L., and C.H.W. approved final version of manuscript.

## APPENDIX

### Integration by Parts

*Equation 1* multiplied with a test function *v* ∈ *S* and integrated by parts leads to

(22) $$-\underset{\Omega }{\int }\left(\nabla \cdot {\overset{\to }{j}}^{P}\right)vdV=-\underset{\Omega }{\int }\nabla \cdot \left(\sigma \nabla u\right)vdV=\underset{\Omega }{\int }\sigma \nabla u\cdot \nabla vdV-\underset{\partial \Omega }{\int }\left(\sigma \frac{\partial u}{\partial n}\right)vdS$$

The last term on the right hand side can be written as follows:

(23) $$-\underset{\partial \Omega }{\int }\left(\sigma \frac{\partial u}{\partial n}\right)vdS=-\overset{L}{\underset{\ell =1}{\sum }}\underset{e_{\ell }}{\int }\left(\sigma \frac{\partial u}{\partial n}\right)vdS\;=-\overset{L}{\underset{\ell =1}{\sum }}\frac{1}{Z_{\ell }A_{\ell }}\underset{e_{\ell }}{\int }\left(U_{\ell }-u\right)vdS$$

This can be expanded as $\overset{L}{\underset{\ell =1}{\sum }}\frac{U_{\ell }}{{(Z}_{\ell }A_{\ell })}\int_{e_{\ell }}^{}vdS-\overset{L}{\underset{\ell =1}{\sum }}\frac{1}{{(Z}_{\ell }A_{\ell })}\int_{e_{\ell }}uvdS.{}_{}$ Because *U*_*ℓ*_ is the integral average of *u* over the electrode surface, it holds that

(24) $$-\underset{e_{\ell }}{\int }\left(\sigma \frac{\partial u}{\partial n}\right)v\;dS=-\overset{L}{\underset{\ell =1}{\sum }}\frac{1}{Z_{\ell }A_{\ell }}\underset{e_{\ell }}{\int }uv\;dS+\sum \frac{1}{Z_{\ell }A_{\ell }^{2}}\underset{e_{\ell }}{\int }u\;dS\underset{e_{\ell }}{\int }v\;dS$$

The final form (*Eq. 24*) substituted into *Eq. 22* yields *Eq. 5*.

### IAS Method

The IAS algorithm (Calvetti et al. 2009) described in *Minimum Current Estimation* refers to alternating conditional minimization of the function

(25) $$F\left(x,t\right)={\left\|Lx-y\right\|}_{2}^{2}+\alpha {\left\|diag{\left(t\right)}^{-1}x\right\|}_{2}^{2}+\overset{M}{\underset{j=1}{\sum }}t_{j}={\left\|Lx-y\right\|}_{2}^{2}+\alpha \overset{M}{\underset{j=1}{\sum }}\frac{x_{j}^{2}}{t_{j}}+\alpha \overset{M}{\underset{j=1}{\sum }}t_{j},$$

where *t*_*j*_ > 0, *j* = 1,2 . . . ,*M*. Because *F*(*x*,*t*) is quadratic, its conditional minimizer *x̃* can be obtained via

(26) $$\overset{˜}{x}={\left(L^{T}L+\alpha \Gamma_{t}\right)}^{-1}L^{T}y,\Gamma_{t}=diag{\left(t\right)}^{-1}\;and\;\Gamma_{0}=I.$$

Furthermore, the gradient of *F*(*x*|*t*) is zero at the conditional minimizer *t̃* of the function *F*(*x*|*t*), i.e.,

(27) $$\frac{\partial F\left(t|x\right)}{\partial t_{j}}|{}_{\overset{˜}{t}}=-\alpha \frac{x_{j}^{2}}{{\overset{˜}{t}}_{j}^{2}}+1=0,\;i.e.,\;\overset{˜}{t}=\left|x_{j}\right|\sqrt{\alpha }$$

With respect to *x*, the IAS algorithm (in *Minimum Current Estimation*) is identical to the following alternating minimization process:

1) Set *t*_0_ = (1,1, . . . ,1) and *l* = 1. For a desired number of iterations repeat the following two steps.
2) Find the conditional minimizer *x̃* of the function *F*(*tt̄*_l−1_).
3) Find the conditional minimizer *t̃* of the function *F*(*t*|*x*_*l*_).

If a global minimizer of *F* is obtained through this iteration, then (*t*)_*j*_ = ||*x*_*j*_||, and it holds that

(28) $$F\left(x,t\right)={\left\|Lx-y\right\|}_{2}^{2}+\alpha \overset{M}{\underset{j=1}{\sum }}\frac{x_{j}^{2}}{\left|x_{j}\right|\sqrt{\alpha }}+\overset{M}{\underset{j=1}{\sum }}t_{j}\sqrt{\alpha }={\left\|Lx-y\right\|}_{2}^{2}+2\sqrt{\alpha }{\left\|x\right\|}_{1}$$

The final form indicates that the global minimizer (*x,t*) is also the 1-norm regularized solution of the linearized inverse problem, i.e., a minimum current estimate (MCE) with a minimal number of nonzero entries.

## References

[B1] Acar ZA, Makeig S Neuroelectromagnetic forward head modeling toolbox. J Neurosci Methods 190: 258–270, 2010. 20457183 10.1016/j.jneumeth.2010.04.031 PMC4126205

[B2] American Clinical Neurophysiology Society. Guideline 3: minimum technical standards for EEG recording in suspected cerebral death. J Clin Neurophysiol 23: 97, 2006. 16612224 10.1097/00004691-200604000-00004

[B3] Awada K, Jackson D, Williams J, Wilton D, Baumann S, Papanicolaou A Computational aspects of finite element modeling in EEG source localization. IEEE Trans Biomed Eng 44: 736–751, 1997. 9254987 10.1109/10.605431

[B4] Baillet S, Mosher JC, Leahy RM Electromagnetic brain mapping. IEEE Signal Process Mag 18: 14–30, 2001.

[B5] Bauer M, Pursiainen S, Vorwerk J, Köstler H, Wolters CH Comparison study for whitney (Raviart-Thomas) type source models in finite element method based EEG forward modeling. IEEE Trans Biomed Eng 62: 2648–2656, 2015. 26054057 10.1109/TBME.2015.2439282

[B6] Baumann S, Wozny D, Kelly S, Meno F The electrical conductivity of human cerebrospinal fluid at body temperature. IEEE Trans Biomed Eng 44: 220–223, 1997. 9216137 10.1109/10.554770

[B7] Braess D Finite Elements. Cambridge, MA: Cambridge Univ Press, 2001.

[B8] Breitling C, Zaehle T, Dannhauer M, Bonath B, Tegelbeckers J, Flechtner HH, Krauel K Improving interference control in ADHD patients with transcranial direct current stimulation (tDCS). Front Cell Neurosci 10: 72, 2016. 27147964 10.3389/fncel.2016.00072 PMC4834583

[B9] Buchner H, Knoll G, Fuchs M, Rienacker A, Beckmann R, Wagner M, Silny J, Pesch J Inverse localization of electric dipole current sources in finite element models of the human head. Electroencephalogr Clin Neurophysiol 102: 267–278, 1997. 9146486 10.1016/s0013-4694(96)95698-9

[B10] Calvetti D, Hakula H, Pursiainen S, Somersalo E Conditionally gaussian hypermodels for cerebral source localization. SIAM J Imag Sci 2: 879–909, 2009.

[B11] Cheney M, Isaacson D, Newell JC Electrical impedance tomography. SIAM Rev 41: 85–101, 1999.

[B12] Cheng KS, Isaacson D, Newell J, Gisser D Electrode models for electric current computed tomography. Biomed Eng IEEE Trans 36: 918–924, 1989. 10.1109/10.35300 PMC4816634 2777280

[B13] Cook M, Koles Z A high-resolution anisotropic finite-volume head model for EEG source analysis In: Proc. 28th Ann Int Conf IEEE Eng Med Biol Soc, p. 4536–4539, 2006. 10.1109/IEMBS.2006.260314 17947096

[B14] Dale AM, Fischl B, Sereno MI Cortical surface-based analysis: I. segmentation and surface reconstruction. Neuroimage 9: 179–194, 1999. 9931268 10.1006/nimg.1998.0395

[B15] Dannhauer M, Lanfer B, Wolters C, Knösche T Modeling of the human skull in EEG source analysis. Hum Brain Mapp 32: 1383–1399, 2011. 20690140 10.1002/hbm.21114 PMC6869856

[B16] Dannhauer M, Brooks D, Tucker D, MacLeod R A pipeline for the simulation of transcranial direct current stimulation for realistic human head models using scirun/biomesh3D. Conf Proc Ann Int Conf IEEE Eng Med Biol Soc IEEE Eng Med Biol Soc Conf 2012, 2012. 10.1109/EMBC.2012.6347236 PMC3651514 23367171

[B17] de Munck J, Wolters C, Clerc M 2012 EEG & MEG forward modeling. In: Handbook of Neural Activity Measurement, edited by Brette R, Destexhe A New York, NY: Cambridge Univ Press, 2012.

[B18] Duffy FH, Iyer VG, Surwillo WW Clinical Electroencephalography and Topographic Brain Mapping: Technology and Practice. New York, NY: Springer, 2012.

[B19] Eichelbaum S, Dannhauer M, Hlawitschka M, Brooks D, Knösche TR, Scheuermann G Visualizing simulated electrical fields from electroencephalography and transcranial electric brain stimulation: A comparative evaluation. Neuroimage 101: 513–530, 2014. 24821532 10.1016/j.neuroimage.2014.04.085 PMC4172355

[B20] Gargiulo P, Belfiore P, Friethgeirsson EA, Vanhatalo S, Ramon C The effect of fontanel on scalp EEG potentials in the neonate. Clin Neurophysiol 126: 1703–1710, 2015. 25553851 10.1016/j.clinph.2014.12.002

[B21] Gramfort A, Papadopoulo T, Olivi E, Clerc M Forward field computation with openMEEG. Comput Intell Neurosci 2011: 1–13, 2011. 21437231 10.1155/2011/923703 PMC3061324

[B22] Guler S, Dannhauer M, Erem B, Macleod R, Tucker D, Turovets S, Luu P, Erdogmus D, Brooks DH Optimization of focality and direction in dense electrode array transcranial direct current stimulation (tDCS). J Neur Eng 13: 036020, 2016a. 10.1088/1741-2560/13/3/036020 PMC5198846 27152752

[B23] Guler S, Dannhauer M, Erem B, Macleod R, Tucker D, Turovets S, Luu P, Meleis W, Brooks D Optimizing stimulus patterns for dense array tDCS with fewer sources than electrodes using a branch and bound algorithm. In: Biomed Imaging (ISBI) 2016 IEEE Int Symp, 2016b. 10.1109/ISBI.2016.7493251 PMC5417550 28479959

[B24] Haueisen J Methods of numerical field calculation for neuromagnetic source localization (PhD thesis). Herzogenrath, Germany: Shaker-Verlag Aachen, ISBN 3–8265-1691-5, 1996.

[B25] Herrmann CS, Rach S, Neuling T, Strüber D Transcranial alternating current stimulation: a review of the underlying mechanisms and modulation of cognitive processes. Front Hum Neurosci 7: 279, 2013. 23785325 10.3389/fnhum.2013.00279 PMC3682121

[B26] Lau S, Güllmar D, Flemming L, Grayden DB, Cook MJ, Wolters CH, Haueisen J Skull defects in finite element head models for source reconstruction from magnetoencephalography signals. Front Neurosci 10: 141, 2016. 27092044 10.3389/fnins.2016.00141 PMC4823312

[B27] Lew S, Sliva DD, Choe MS, Grant PE, Okada Y, Wolters CH, Hämäläinen MS Effects of sutures and fontanels on MEG and EEG source analysis in a realistic infant head model. NeuroImage 76: 282–293, 2013. 23531680 10.1016/j.neuroimage.2013.03.017 PMC3760345

[B28] Lew S, Wolters CH, Anwander A, Makeig S, MacLeod RS Improved EEG source analysis using low-resolution conductivity estimation in a four-compartment finite element head model. Hum Brain Map 30: 2862–2878, 2009. 10.1002/hbm.20714 PMC2733918 19117275

[B29] Liehr M, Haueisen J Influence of anisotropic compartments on magnetic field and electric potential distributions generated by artificial current dipoles inside a torso phantom. Phys Med Biol 53: 245, 2007. 18182700 10.1088/0031-9155/53/1/017

[B30] Lucka F, Pursiainen S, Burger M, Wolters CH Hierarchical Bayesian inference for the EEG inverse problem using realistic FE head models: depth localization and source separation for focal primary currents. NeuroImage 61: 1364–1382, 2012. 22537599 10.1016/j.neuroimage.2012.04.017

[B31] Montes-Restrepo V, van Mierlo P, Strobbe G, Staelens S, Vandenberghe S, Hallez H Influence of skull modeling approaches on EEG source localization. Brain Topogr 27: 95–111, 2014. 24002699 10.1007/s10548-013-0313-y

[B32] Mosher JC, Leahy RM, Lewis PS EEG/MEG: Forward solutions for inverse methods. IEEE Transact Biomed Eng 46: 245–259, 1999. 10.1109/10.748978 10097460

[B33] Niedermeyer E, da Silva FL Electroencephalography: Basic Principles, Clinical Applications, and Related Fields (5th ed). Philadelphia, PA: Lippincott Williams & Wilkins, 2004.

[B34] Odabaee M, Tokariev A, Layeghy S, Mesbah M, Colditz PB, Ramon C, Vanhatalo S Neonatal EEG at scalp is focal and implies high skull conductivity in realistic neonatal head models. NeuroImage 96: 73–80, 2014. 24736169 10.1016/j.neuroimage.2014.04.007

[B35] Ollikainen J, Vauhkonen M, Karjalainen PA, Kaipio JP Effects of electrode properties on EEG measurements and a related inverse problem. Med Eng Phys 22: 535–545, 2000. 11182578 10.1016/s1350-4533(00)00070-9

[B36] Pursiainen S Raviart-Thomas-type sources adapted to applied EEG and MEG: implementation and results. Inverse Problems 28: 065013, 2012.

[B37] Pursiainen S, Sorrentino A, Campi C, Piana M Forward simulation and inverse dipole localization with lowest order Raviart-Thomas elements for electroencephalography. Inverse Problems 27: 045003, 2011.

[B38] Schönen R, Rienäcker A, Beckmann R, Knoll G 1994 Dipolabbildung im FEM-Netz, Teil I Arbeitspapier zum Projekt Anatomische Abbildung elektrischer Aktivität des Zentralnervensystems, R.W.T.H. Aachen, Germany, 1994.

[B39] Somersalo E, Cheney M, Isaacson D Existence and uniqueness for electrode models for electric current computed tomography. SIAM J Appl Math 52: 1023–1040, 1992.

[B40] Stenroos M, Sarvas J Bioelectromagnetic forward problem: isolated source approach revis(it)ed. Phys Med Biol 57: 3517–3535, 2012. 22581305 10.1088/0031-9155/57/11/3517

[B41] Tanzer O, Järvenpää S, Nenonen J, Somersalo E Representation of bioelectric current sources using Whitney elements in the finite element method. Phys Medicine Biol 50: 3023–3039, 2005. 10.1088/0031-9155/50/13/004 15972978

[B42] Toupin R Saint-Venant's principle. Arch Ration Mech Anal 18: 83–96, 1965.

[B43] Uutela K, Hämäläinen M, Somersalo E Visualization of magnetoencephalographic data using minimum current estimates. NeuroImage 10: 173–180, 1999. 10417249 10.1006/nimg.1999.0454

[B44] Vallaghé S, Papadopoulo T, Clerc M The adjoint method for general EEG and MEG sensor-based lead field equations. Phys Medicine Biol 54: 135, 2009. 10.1088/0031-9155/54/1/009 19075359

[B45] Vatta F, Meneghini F, Esposito F, Mininel S, Salle FD Solving the forward problem in EEG source analysis by spherical and FDM head modeling: a comparative analysis. Biomed Sci Instrum 45: 382–388, 2009. 19369793

[B46] Vauhkonen M Electrical Impedance Tomography and Prior Information (PhD thesis) Kuopio, Finland: University of Kuopio, 1997.

[B47] Weinstein D, Zhukov L, Johnson C Lead-field bases for electroencephalography source imaging. Ann Biomed Eng 28: 1059–65, 2000. 11132189 10.1114/1.1310220

[B48] Wendel K, Narra N, Hannula M, Kauppinen P, Malmivuo J The influence of CSF on EEG sensitivity distributions of multilayered head models. IEEE Trans Biomed Eng 55: 1454–1456, 2008. 18390339 10.1109/TBME.2007.912427

[B49] Wetterling F, Liehr M, Schimpf P, Liu H, Haueisen J The localization of focal heart activity via body surface potential measurements: tests in a heterogeneous torso phantom. Phys Med Biol 54: 5395, 2009. 19700819 10.1088/0031-9155/54/18/003

[B50] Yan Y, Nunez PL, Hart RT Finite-element model of the human head: scalp potentials due to dipole sources. Med Biol Eng Comput 29: 475–481, 1991. 1817208 10.1007/BF02442317

